# Safety of an Oncolytic Myxoma Virus in Dogs with Soft Tissue Sarcoma

**DOI:** 10.3390/v10080398

**Published:** 2018-07-28

**Authors:** Amy L. MacNeill, Kristen M. Weishaar, Bernard Séguin, Barbara E. Powers

**Affiliations:** 1 Department of Microbiology, Immunology and Pathology, College of Veterinary Medicine and Biomedical Sciences, Colorado State University, Fort Collins, CO 80523, USA; 2 Department of Clinical Sciences, College of Veterinary Medicine and Biomedical Sciences, Colorado State University, Fort Collins, CO 80523, USA; Kristen.Weishaar@colostate.edu (K.M.W.); Bernard.Seguin@colostate.edu (B.S.); 3 Veterinary Diagnostic Laboratories, Colorado State University, Fort Collins, CO 80523, USA; Barb.Powers@colostate.edu

**Keywords:** oncolytic poxvirus, myxoma virus, sarcoma, canine

## Abstract

Many oncolytic viruses that are efficacious in murine cancer models are ineffective in humans. The outcomes of oncolytic virus treatment in dogs with spontaneous tumors may better predict human cancer response and improve treatment options for dogs with cancer. The objectives of this study were to evaluate the safety of treatment with myxoma virus lacking the *serp2* gene (MYXVΔserp2) and determine its immunogenicity in dogs. To achieve these objectives, dogs with spontaneous soft tissue sarcomas were treated with MYXVΔserp2 intratumorally (*n* = 5) or post-operatively (*n* = 5). In dogs treated intratumorally, clinical scores were recorded and tumor biopsies and swabs (from the mouth and virus injection site) were analyzed for viral DNA at multiple time-points. In all dogs, blood, urine, and feces were frequently collected to evaluate organ function, virus distribution, and immune response. No detrimental effects of MYXVΔserp2 treatment were observed in any canine cancer patients. No clinically significant changes in complete blood profiles, serum chemistry analyses, or urinalyses were measured. Viral DNA was isolated from one tumor swab, but viral dissemination was not observed. Anti-MYXV antibodies were occasionally detected. These findings provide needed safety information to advance clinical trials using MYXVΔserp2 to treat patients with cancer.

## 1. Introduction

There is a growing interest in using viruses to eliminate cancers. In fact, the first oncolytic virus approved for use in the United States was marketed in October 2015 for treatment of melanoma in humans [1]. However, more robust progress in this field has been hampered by the fact that murine cancer models are poor predictors of oncolytic virus efficacy in human cancer patients. Evaluation of cancer treatments in dogs with spontaneous tumors has gained acceptance as a model for human disease, but trials using oncolytic viruses to treat canine cancer patients are rare [2]. Many publications indicate that oncolytic viruses can replicate and lyse canine cancer cells in culture [2,3,4,5,6,7,8,9,10]. In addition, murine xenograft models of canine cancers have been successfully treated with oncolytic viruses [3,5,6,7,10,11,12,13,14,15,16,17,18,19,20,21,22,23]. There have been several studies that indicate that oncolytic viral therapy is safe to use in dogs [4,5,11,24,25,26,27,28,29,30,31,32]. Clinical trials using oncolytic virotherapy in dogs are the next step in the process of advancing treatment options for dogs with cancer. Simultaneously, these studies may provide a more accurate predictive model of human response to virotherapy [33].

In order for oncolytic virotherapy to be an effective treatment modality, the virus must be able to selectively enter, replicate in, and lyse tumor cells [34]. At the same time, the virus must be nonpathogenic. Poxviruses have several innate characteristics that make them promising oncolytic viruses. For example, although a specific cellular receptor is not needed for poxviruses to infect cells [35], they naturally target areas of neovascularization within neoplasms [36]. Poxviruses are effective vectors for exogenous protein expression due to their large, linear, double-stranded DNA genomes, which replicate with high fidelity and allow for extensive genetic modification [37]. Importantly, unlike some of the other oncolytic viruses, poxviral DNA remains in the cytoplasm of host cells and cannot inadvertently recombine into the host DNA. Once a poxvirus enters a host cell, transcription begins quickly and virions are released before cell death occurs. This allows for efficient spread of virus within the tumor microenvironment [6,36]. Additionally, poxviruses are rapidly cleared from the body because they incite strong cell-mediated and humoral immune responses which prevent latent or recurrent infections from occurring [38,39,40,41,42].

There is evidence that a host-restricted poxvirus, myxoma virus (MYXV), can be used as a safe, effective cancer treatment. MYXV does not cause disease in humans or other vertebrates, with the exception of rabbits [32,43,44,45,46,47,48,49]. In spite of its species specificity, MYXV productively infects cultured cancer cells from several animal species [8,50,51,52]. In rodent cancer models, MYXV treatment has eliminated some glioma xenografts and reduced tumor burden in several types of xenografts and allografts [53,54,55,56,57,58,59,60,61,62,63,64,65,66,67]. A recombinant MYXV, MYXV deleted for *serp2* (MYXVΔserp2) was chosen for use in this study. Serp2 is an anti-apoptotic protein and a virulence factor; deletion of this protein from the virus enhances the oncolytic effects of the virus and markedly attenuates pathogenesis in rabbits [8,68]. The data presented in this manuscript support our hypothesis that injection of MYXVΔserp2 is safe in dogs with soft tissue sarcomas (STS). Results indicate that further evaluation of the oncolytic effects of MYXVΔserp2 in cancer patients is warranted.

## 2. Materials and Methods 

### 2.1. Recombinant MYXVΔserp2

Nathaniel et al. described construction of recombinant MYXVΔserp2 (MYXVΔserp2::lacZ) and its decreased virulence in rabbits [69]. In another study, the same MYXVΔserp2 construct was shown to induce increased cytopathic effects in canine cancer cells as compared to wild-type MYXV [8]. For these trials, MYXVΔserp2 constructed in the Moyer laboratory [69] was sucrose pad purified, titered, and diluted to 10^6^ plaque-forming units (pfu) of MYXVΔserp2 per mL phosphate-buffered saline (PBS). This dose was based upon a rhabdomyosarcoma xenograft model in which multiple doses of 10^6^ pfu of MYXV significantly improved outcome in mice [59].

### 2.2. Treatment with MYXVΔserp2

Use of MYXV∆serp2 was approved by the Colorado State University (CSU) Institutional Biosafety Committee (IBC #14-026B, 5/7/2015). Intratumoral MYXV∆serp2 treatment of the five dogs enrolled in the first arm of the study was approved by the CSU Institutional Animal Care and Use Committee (IACUC #15-5737A, 5/4/2015). Post-operative MYXV∆serp2 treatment of the five dogs enrolled in the second arm of the study was approved by the CSU Veterinary Teaching Hospital Clinical Review Board (Veterinary Clinical Studies #2016-061, 7/11/2016) and by IBC #16-073B, 9/28/2017).

#### 2.2.1. Patient Enrollment

The Clinical Trials Team at CSU Flint Animal Cancer Center (directed by a board-certified veterinary oncologist, K.M.W.) coordinated entry of the patients into the study, obtained informed client consent, and scheduled appointment dates and times. For dogs in the intratumoral treatment arm of the study, patients were required to have a histologically confirmed sarcoma that was at least 2 cm in diameter and accessible for biopsy. To be enrolled in the post-operative treatment arm of the study, dogs had to have a histologically confirmed grade 2 or 3 sarcoma with a longest diameter ≤ 12 cm that was deemed not amenable to complete surgical resection. Inclusion criteria for both study arms also included agreement from the owner that no chemotherapy, radiation therapy, or other anti-cancer treatment would be administered until the tumor progressed or recurred, metastatic disease was detected, or after completion of this study. Prior chemotherapy and/or radiation therapy were allowed with specified washout periods. Adequate organ function was required as indicated by standard laboratory tests (specifically, neutrophils > 2000 cells/µL; hematocrit > 30%; platelets > 75,000/µL; creatinine < 2× the upper reference limit; bilirubin ≤ 1.5× the upper reference limit. Owners were not able to enroll their pet if any immunocompromised people lived in the household or if anyone in the household owned a rabbit.

#### 2.2.2. Intratumoral Injection of MYXVΔserp2

Five dogs diagnosed with subcutaneous sarcoma by histopathologic evaluation of biopsies taken at CSU were deemed eligible for the intratumoral treatment arm of the study. Once enrolled, dogs were treated with a single intratumoral injection of 10^6^ pfu purified MYXVΔserp2 diluted in 1 mL PBS. The skin surface at the injection site was disinfected with an accelerated hydrogen peroxide solution (Accel Disinfectant Wipe, Virox Technologies, Oakville, ON, Canada) to remove any residual virus from the patients’ skin. The day of treatment was designated as Day 0. Veterinarians on the Clinical Trials Team extensively monitored the first two dogs daily for seven days and then weekly until Day 28 following intratumoral inoculation. The third dog was examined by the Clinical Trials Team on Days 0–4, 7, 14, 21, and 30. The last two dogs were evaluated by the Clinical Trials Team on Days 0, 1, 4, 7, and then weekly until Day 28 or 29.

#### 2.2.3. Post-Operative Injection of MYXVΔserp2

Five dogs afflicted with histologically confirmed grade 2 or grade 3 STS that were not amenable to a wide excision because of size and anatomic location (and therefore likely to be incompletely excised) were enrolled in the post-operative treatment arm of the study. Tumors were marginally excised by a board-certified veterinary surgeon (B.S.) and tumor margins were marked and evaluated histologically. Two MYXVΔserp2 treatments were administered in attempt to treat residual disease at the surgery site. For the first treatment, the perimeter of the surgical site was inoculated with 5–10 doses of 10^6^ pfu purified MYXVΔserp2 diluted in 1 mL PBS. Injections were administered at 2 cm intervals around and 5 mm from the edges of the surgical margins so that the number of virus particles injected per surface area of the surgical site remained uniform. The skin surface at the surgical site was disinfected with an accelerated hydrogen peroxide solution. The day of surgery was designated as Day 0. For the second treatment, the virus inoculation and disinfection of the skin surface were repeated at the time of suture removal, approximately two weeks after the initial treatment. If the surgical site was not healed or there was a seroma present two weeks post-surgery, the virus injections were delayed an additional two weeks. The second virus treatment was an attempt to boost the immune response to the virus during the course of the trial. Appendix A, Table A1 indicates the total amount of virus administered to each patient.

### 2.3. Patient Monitoring

In the dogs given an intratumoral injection, medical monitoring of treatment effects included daily measurement of the tumor using calipers, measurement of patient weight, and scoring of clinical signs for 28–30 days after MYXVΔserp2 treatment. The clinical scoring system used for this study is provided in Appendix A (Table A2). Each day, the clinical scores were added together and the change in the total daily score as compared to Day 0 (immediately before MYXVΔserp2 injection) was calculated. For dogs given an intratumoral injection of MYXVΔserp2, blood, urine, feces, and swabs from the mouth and the site of virus injection were collected prior to MYXVΔserp2 treatment and on post-treatment Days 0–7, 14, 21, and 28 for the first two dogs, on Days 0–4, 7, 14, 21, and 30 for the third dog, and on Days 0, 1, 4, 7, 14, 21, and 28 or 29 for the last two dogs. In the dogs injected with MYXVΔserp2 post-operatively, blood, urine, and feces were collected prior to surgery, at the time of suture removal, and two weeks after suture removal. Response to therapy was determined according to the veterinary response evaluation criteria for solid tumors (RECIST) in dogs [70]. Adverse events (AE) were graded according to the Veterinary Comparative Oncology Group common terminology criteria for AE (VCOG-CTCAE) [71].

At the time-points indicated in the previous paragraph, whole blood (collected into a potassium ethylenediaminetetraacetic acid blood collection tube) and serum were submitted to the Clinical Pathology Laboratory at CSU for a complete blood count (parameters evaluated included absolute cell numbers, erythrocyte hematocrit (Hct), clinically important cellular indices (e.g., hemoglobin content, mean cell volumes), and microscopic evaluation of erythrocytes, leukocytes, and platelets) and serum chemistry profile (parameters measured included glucose, blood urea nitrogen (BUN), creatinine, calcium, magnesium, total protein, albumin, cholesterol, total bilirubin, alkaline phosphatase (ALP), alanine aminotransferase (ALT), aspartate aminotransferase (AST), creatine kinase (CK), gamma-glutamyl transferase (GGT), sodium, potassium, chloride, and bicarbonate; calculated values included globulins, albumin to globulin ratio, and anion gap). A urinalysis also was performed. Parameters recorded for urine included color, clarity, specific gravity by refractometry, reagent pad colometric changes (for pH, nitrite, protein, glucose, ketones, bilirubin concentrations, and blood), and microscopic sediment analysis. These data were evaluated by a board-certified veterinary clinical pathologist (A.L.M.) to assess the overall health of the patients.

### 2.4. Detection of Viral DNA

Samples used to determine if MYXVΔserp2 spread from the injection site were collected at time-points indicated in Section 2.3. Blood, urine, and feces from all 10 patients, and swabs from the mouth and the surface of the tumor of five patients injected intratumorally were tested for MYXVΔserp2 DNA. DNA was extracted from the samples using a DNeasy Blood and Tissue Kit (Qiagen Sciences, Germantown, MD, USA). Positive control samples for PCR included DNA from purified MYXV DNA and DNA extracted from a swab dipped into purified MYXV DNA. The negative control sample included diethyl pyrocarbonate treated water instead of DNA. On Days 4 and 14, tumor biopsies were taken from the dogs that were injected with MYXVΔserp2 intratumorally. One half of the biopsy section was homogenized and DNA was extracted using a Qiagen Kit for detection of viral DNA using PCR. Two sets of PCR primers were designed to amplify different regions of the MYXVΔserp2 genome (Table 1). A standard 35 cycle PCR reaction protocol was used. Any questionable or positive PCR results were quantified by droplet digital PCR (ddPCR) using a QX200 Droplet Digital PCR System (Bio-Rad, Hercules, CA, USA). For the ddPCR assay, portions of myxoma virus DNA polymerase and canine glyceraldehyde 3-phosphate dehydrogenase (*cGAPDH*) genes were targeted. The *cGAPDH* amplification was used as an internal control to ensure adequate DNA was present in each canine sample. We determined that this assay could detect as few as 10 MXYV∆serp2 viral genomes in a 1 mg section of skin. Primer sequences are listed in Table 1.

### 2.5. Evaluation of the Immune Response to MYXVΔserp2

Basic aspects of the innate and adaptive immune responses (including tumor inflammation, leukocyte activation, and antibody production) were evaluated using histopathology, flow cytometry, and virus neutralization assays to determine if the immune system of dogs was activated by MYXVΔserp2 treatment.

#### 2.5.1. Tumor Histopathology

Samples were submitted to the Histopathology Section of the Colorado State University Veterinary Diagnostic Laboratories for processing and Hematoxylin and Eosin staining. Subsections of pre-treatment, and Day 4 and 14 post-treatment biopsies collected from dogs that were injected with MYXVΔserp2 intratumorally were evaluated for tumor grade, mitoses, necrosis, and immune cell infiltrates into the tumors. In dogs treated with MYXVΔserp2 post-operatively, pre-surgical biopsy samples were evaluated for tumor grade and the presence of inflammation. Surgically excised tumor sections were assessed for residual disease (tumor cells that extended to the margins of the excised tissue). A board certified veterinary anatomic pathologist (B.E.P.) examined all tumor samples.

#### 2.5.2. Flow Cytometry of Peripheral Blood Leukocytes

Whole blood samples were submitted to the Clinical Immunology Laboratory at CSU to identify subsets of leukocytes using flow cytometry. The percentages of subsets of B-lymphocytes (characterized by detection of CD21 and MHC II cell membrane proteins), T-lymphocytes (characterized by CD3, CD4, CD8, and CD25), and monocytes (characterized by CD14, CD18, and MHC II) were determined using Kaluza Analysis Software Version 1.3 (Beckman Coulter Life Sciences, Indianapolis, IN, USA).

#### 2.5.3. Anti-MYXV Antibody Response

Serum was evaluated for neutralizing antibodies against MYXV using a plaque reduction neutralization test (PRNT) as previously described [54,61]. Briefly, serum was serially diluted and incubated with MYXV (multiplicity of infection = 1) in 24-well cell culture plates containing confluent rabbit-kidney epithelial (RK-13) cell monolayers. Infected cells were collected and serial dilutions were made. Confluent RK-13 cells in 6-well plates were inoculated with dilutions of infected cells and then overlaid with a solid media to cause infectious virus to form foci on cell monolayers. Virus titers (pfu/mL of diluted sample) were determined and plotted versus the reciprocal of the sample dilution of serum. Using Microsoft Excel^®^ 2010 (Microsoft Corporation, Redmond, WA, USA), the sample dilution of the 50% maximum virus titer endpoint was determined by linear interpolation. Serum collected from rabbits 10–14 days after MYXV inoculation was used as a positive control for the presence of neutralizing antibodies.

## 3. Results

The primary aim of this study was to determine if injection of purified MYXVΔserp2 was safe in dogs with subcutaneous sarcoma. Patient demographics are described below (3.1) and are summarized in Table 2. Both patient safety and environmental safety were considered. Patient safety was assessed by closely monitoring the health of the patients using clinical data (3.2). Environmental safety was evaluated using PCR to determine if virus was shed from the dogs after treatment (3.3).

The second aim of this study was to analyze the basic immune response to MYXVΔserp2 treatment in dogs with STS. Histopathology of the tumor (3.4), flow cytometry of peripheral blood leukocytes (3.5), and detection of neutralizing anti-MYXV antibodies in sera (3.6) were performed to characterize the effect of MYXVΔserp2 injection on the canine immune system.

### 3.1. Patient Demographics

Ten dogs with a histologic diagnosis of sarcoma were recruited into this study. The patients were between 8 and 15 years of age (median = 11 years). Breeds enrolled in the study included a miniature schnauzer, an Italian greyhound, a Shetland sheepdog, a greyhound, two Labrador retrievers, and four mixed-breed dogs. Five of ten dogs had experienced tumor regrowth at the site of surgical removal of a sarcoma before enrolling in this study. All five dogs given an intratumoral injection of MYXVΔserp2 were castrated male dogs. Three of five dogs given post-operative injections of MYXVΔserp2 were spayed females and two were castrated males.

### 3.2. Clinical Data

Health of the patients was assessed using clinical data including: (1) physical examination, (2) clinical score, (3) body weight, (4) tumor diameter, (5) compete blood counts, (6) serum biochemistry, and (7) urinalysis.

Physical examinations were performed each day that the dogs were at the CSU Veterinary Teaching Hospital (6 to 11 examinations, depending on the timing of patient enrollment). Largest tumor diameter data, tumor location, and tumor biopsy diagnosis are listed in Table 2 for each patient. Additional abnormalities on physical exam at the time of study enrollment included mildly enlarged regional lymph nodes (Dog 3), a heart murmur (Dogs 1, 2, and 7), and one or more benign subcutaneous masses (Dogs 2, 4, 5, 6, 7, 8, and 9). Comorbidities were present in two dogs; Dog 2 had a history of renal disease with associated hypertension and proteinuria, and Dog 9 had previously been diagnosed with bilateral laryngeal paralysis, suspected degenerative axonopathy, and bilateral coxofemoral and elbow osteoarthritis.

**Figure 1:**
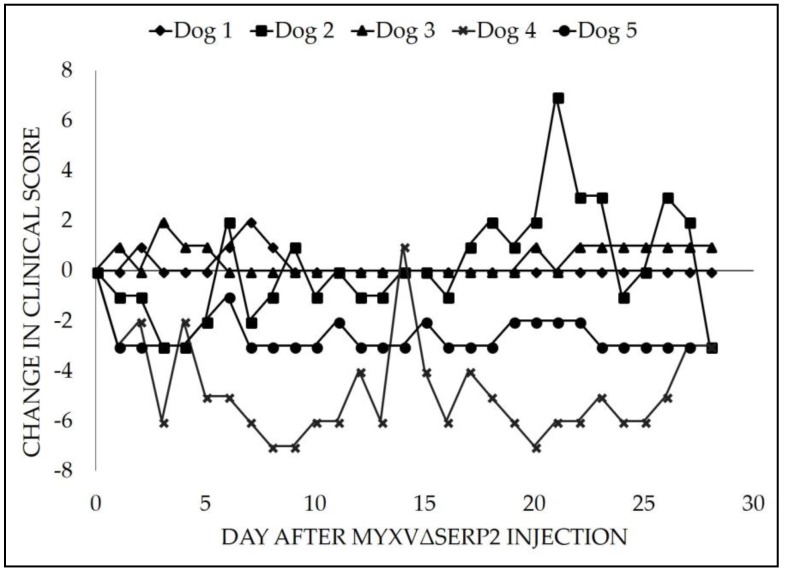
Change in clinical score after an intratumoral injection of 10^6^ pfu MYXVΔserp2 in dogs with spontaneously arising soft tissue sarcomas.

Clinical scores were recorded daily in dogs treated with MYXVΔserp2 intratumorally as a way to detect any complications that were observed by owners on the days that CSU Clinical Trials Team veterinarians did not directly examine the patients. Most of the individual clinical signs evaluated by the score sheet (Table A2) did not occur (Score = 0) or occurred once and did not require any treatment (Score = 1). Two of five dogs (Dogs 1 and 5) were not assigned any individual clinical scores above 1. At Day 0, CSU Clinical Trials Team veterinarians determined that Dogs 2, 4, and 5 presented with a total summation of clinical scores equal to 3, 7, and 3, respectively, whereas, on Day 0, the total summation of clinical scores for Dogs 1 and 3 was zero. In 3/5 dogs, there were some individual clinical signs that were considered of moderate concern or occurred more than once (Score = 2): licking around the tumor (Dog 2, one day and Dog 4, six days), decreased interest in food (Dogs 2 and 3, one day each), increased urination (Dog 2, three days), diarrhea (Dog 2, two days), anxiety (Dog 2, one day), discharge from the tumor (Dog 4, three days), new mass formation (Dog 4, noted on Day 18), and increased water intake (Dog 4, one day). The new mass that the owner observed on Dog 4 was consistent with swelling and ulceration at the Day 14 biopsy site. No individual clinical signs that were considered severe or required treatment occurred in any patient (Score = 3). No animals were excluded from the study due to side effects. Changes in the summation of clinical scores over time as compared to Day 0 were calculated (Figure 1).

Patients also were evaluated for AEs using VCOG-CTCAE criteria [71]. No AEs were recorded at the injection site of MYXVΔserp2, although a small to medium-sized seroma (without erythema) was observed at the surgical site of Dog 6 on Day 14, which necessitated a two-week delay of administration of the second virus injection and suture removal. Abnormalities recorded on the clinical score sheets (licking, inappetence, polyuria, diarrhea, anxiety, and polydipsia) were mild (Grade 1 AE). The discharge from the tumor site and new mass formation observed in Dog 4 were considered Grade 1 AEs. Additional AEs are discussed in detail below. Appendix A (Table A1) lists all Grade ≥ 2 AE observed during the study.

Minor changes in body weight were calculated during the first month following MYXVΔserp2 treatment. Increased body weight was observed in 2/5 dogs treated with one injection of MYXVΔserp2 and 3/5 dogs given two injections. The remaining 5/10 dogs lost a small amount of weight (Grade 1 AE). The percent change in weight ranged from −9.1% to + 4.6%. The median change in body weight was −2.2%. None of the weight changes were considered unhealthy for the patients.

Complete blood counts were performed to assess hematologic parameters and detect any abnormalities in erythrocytes, leukocytes, or platelets. Complete blood count data were compared to reference intervals for adult dogs established by the Clinical Pathology Laboratory at CSU. Five of 10 dogs had a mild lymphopenia at Day 0. Mild lymphopenia was observed at least once throughout the study in 9/10 dogs. This was attributed to stress, although reactive lymphocytes were noted in blood smears from 3/10 dogs (Dog 1, Days 14, 21, and 28; Dog 3, Days –2, 2, 3, 4, 7, and 14; and Dog 4, Days 0, 14, and 21). Dog 7 had a leukopenia due to neutropenia (Grade 1 AE) and lymphopenia on Day 0 (prior to MYXVΔserp2 treatment); the leukopenia and neutropenia resolved by Day 14, but the lymphopenia remained. Basophils were mildly elevated (Grade 1 AE) in Dog 1 on Days 0 and 14, but were otherwise within reference intervals. Erythrocyte mass, as determined by Hct, was very mildly increased in four patients: Dog 1 (Day 4), Dog 5 (Day 0), Dog 7 (Days 14, 28, and 165), and Dog 10 (Day 0). Most commonly, mildly increased Hct is due to normal biological variability, but mild dehydration also should be considered. A decreased Hct was observed in Dog 2 at all time-points and was attributed to anemia of chronic disease (renal failure). The anemia was classified as a Grade 1 AE on Days 1, 3, 4, 5, and 7 and a Grade 2 AE on Days 2, 6, 14, 21, and 28. All measurements of Hct in the remaining five dogs were within reference intervals. Platelet counts were mildly elevated in two patients: Dog 2 (Days 2, 21, and 28), and Dog 5 (Day 28). In dogs, thrombocytosis is often a stress response or is secondary to inflammation. Mildly decreased (Grade 1 AE) automated platelet counts were observed in three patients: Dog 4 (Day 21), Dog 6 (Day 28), and Dog 7 (Days 0, 14, and 28). A moderately decreased (Grade 2 AE) automated platelet count was recorded for Dog 3 (Day 7). The platelet mass appeared adequate on blood smears at all time-points in all patients with decreased automated platelet counts. Platelet counts were within reference intervals at all time-points in the remaining four patients. Therefore, none of the patients were diagnosed with thrombocytopenia. Overall, complete blood count data were considered stable in all 10 patients but indicated mild stress in 9/10 patients throughout the course of the study.

Serum biochemistry data were collected to evaluate function of many internal organs (e.g., liver, kidney, muscle, etc.). Serum biochemistry data were compared to reference intervals for adult dogs established by the Clinical Pathology Laboratory at CSU. Table A1 lists all Grade ≥ 2 AEs that occurred. The other abnormalities discussed below were Grade 1 AEs [71]. Dog 1 had mildly elevated sodium and chloride concentrations on Day 6, which may indicate mild dehydration, although protein concentrations were within reference intervals throughout the study. Several AEs were observed in Dog 2 who was diagnosed with managed chronic renal failure prior to inclusion in the study. The renal disease was classified as a Grade 2 to 3 throughout the study. This patient had expected moderate increases in markers of kidney function (BUN, creatinine, phosphorus, and potassium concentrations). His calculated anion gap was also mildly elevated on several days, consistent with a mildly increased concentration of uremic acids from renal disease. In Dog 6, total calcium concentration was consistently moderately elevated (Days −2, 14, and 28) but was considered clinically insignificant; therefore, ionized calcium was not evaluated. Mildly decreased magnesium concentration was noted in Dog 7 (Days 0, 14, and 28). This abnormality also was observed on Days −10 and 165 and so was considered normal for this patient. Dog 10 had a mildly elevated glucose concentration on Day 0 prior to treatment; this resolved by Day 15. An insignificant increase in cholesterol concentration (<1.3× the upper reference limit) was observed in Dog 5 (Days 0, 4, and 21) and Dog 9 (Days 0 and 14). Dogs 1, 2, and 9 had clinically insignificant elevations (<1.5× the upper reference limit) in serum concentration of the liver enzyme ALT on several days. AST (derived from liver or muscle) was insignificantly elevated (<1.2× the upper reference limit) in Dog 1 (Days 0, 2, and 7). Total serum ALP was insignificantly to mildly elevated (<2.1× the upper reference limit) in 4/10 patients: Dog 4 in 3 samples (Days 4, 7, and 14) and in Dogs 2, 5, and 8 in all samples. Serum ALP was significantly elevated in Dog 9 at all time-points (Days 0, 14 and 28). These findings were most consistent with increases in the stress-induced isoform of ALP; significant liver disease or bony remodeling were not supported by other clinical findings. No endocrine testing was pursued to further evaluate other potential causes of the elevated ALP concentration in these patients. Creatine kinase, a marker of muscle injury, was increased in 6/10 patients: Dog 1 (Days 0 and 2), Dog 2 (Days 1, 2, 4, 5, and 28), Dog 4 (Day 1), Dog 5 (Days 0, 1, and 28), Dog 6 (Day −2, 14, and 28), and Dog 8 (Days −9 and 32). There were no convincing associations between increased CK concentration and injection of MYXVΔserp2 (Day 0), biopsy (Days 4 and 14 in Dogs 1–5), or tumor removal (Day 0 in Dogs 6–10). No biochemical abnormalities were observed in Dog 3 at any time-point.

Urinalyses were performed to help evaluate renal and urinary tract function. Urine abnormalities were rare except in Dog 2 who had been diagnosed with chronic renal disease before enrollment in the study. Urine specific gravity ranged from 1.007 to 1.015 in this patient and proteinuria was persistent, but a urine protein to creatinine ratio was not performed. Otherwise, the only concerning finding occurred on Day 14 in Dog 6 who had normal numbers of leukocytes in his urine (1–5 per 400× microscopic field), but intracellular bacterial cocci were observed. It is likely that the bacteria were sample contaminants since bacteriuria was not observed on Day 28 even though no treatment was administered.

**Figure 2:**
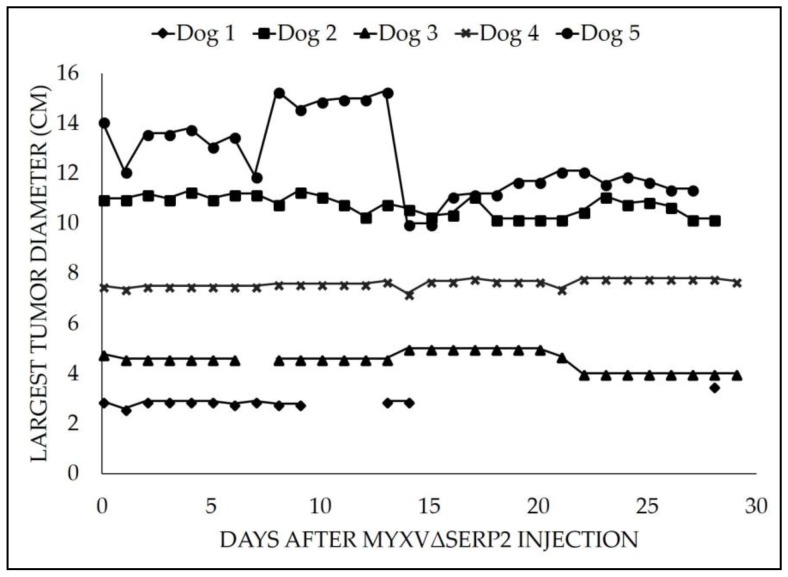
Largest tumor diameter by caliper measurement after an intratumoral injection of 10^6^ pfu MYXVΔserp2 in dogs with spontaneously arising soft tissue sarcomas.

In patients treated with MYXVΔserp2 intratumorally, the maximum tumor diameter (cm) was monitored frequently (Figure 2). Tumor diameter was measured for Dog 1 daily for seven days, then weekly until Day 28. Daily measurements were recorded for Dogs 2, 3, 4, and 5 for 27 to 29 days. Clinically, no convincing evidence of significant growth or regression was observed in 4/5 (80%) patients. A visible reduction in tumor diameter was noted in 1/5 (20%) dogs. At the end of the study, 4/5 dogs had stable disease per the veterinary RECIST criteria, and one had progressive disease [70]. Specifically, Dog 1 had a 29.6% (0.6 cm) increase, Dog 2 had a 7.3% (0.8 cm) decrease, Dog 3 had a 16.7% (0.8 cm) decrease, Dog 4 had a 2.7% (0.2 cm) increase, and Dog 5 had a 19.1% (2.7 cm) decrease in tumor diameter.

In summary, no clinically relevant changes were observed in clinical scores, body weight, complete blood counts, biochemical profiles, or urinalyses from any patient (*n* = 10) during the course of the study. Interestingly, measurement of largest tumor diameter decreased slightly in 3/5 (60%) dogs. Together, the clinical data indicate that up to 10^7^ pfu of purified MYXVΔserp2 is safe to inject subcutaneously either intratumorally or post-operatively in dogs with subcutaneous sarcoma.

### 3.3. Virus Distribution

For the safety of care givers, owners, and the environment, it was critical to ensure that MYXVΔserp2 did not disseminate and shed following intratumoral or post-operative subcutaneous treatment of dogs with STS. Samples tested from all ten dogs included blood, urine, and feces around Days 0, 14, and 28. Samples from the five dogs given an intratumoral injection of MYXVΔserp2 included blood, urine, feces, buccal swabs, and tumor swabs from Days 0–7, 14, 21, and ~28, and tumor biopsy samples from Days 4 and 14. All samples were screened by PCR and then quantified by ddPCR if there was any evidence that they contained viral DNA. Template concentrations obtained using ddPCR indicated that only one patient sample (from Dog 2) contained viral DNA (an average of 37 MYXV and 17 cGAPDH templates/µL). This sample was a Day 0 swab of the tumor surface that was taken immediately after MYXVΔserp2 was injected intratumorally and before the surface was disinfected. For comparison, in the positive control viral DNA sample (with a starting concentration of 1.5 ng/µL), 1321 MYXV and 0 cGAPDH templates/µL were amplified.

### 3.4. Tumor Histopathology

**Figure 3:**
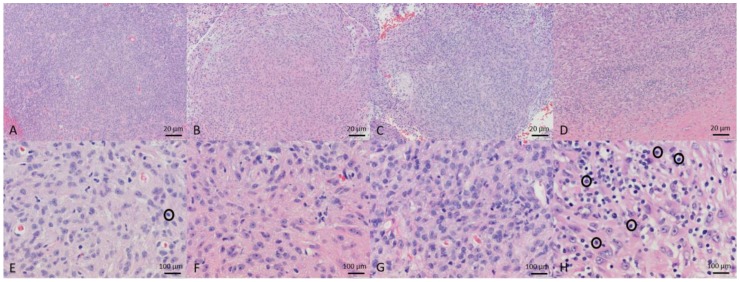
Microscopic images of tissue sections from Dog 5. Biopsy samples were collected before MYXVΔserp2 treatment (**A**,**E**) and on Days 4 (**B**,**F**) and 14 (**C**,**G**) post-treatment. Sections from the tumor resected on Day 31 are also shown (**D**,**H**). Hematoxylin and Eosin stain; ×100 (**A**–**D**) and ×500 (**E**,**F**,**G** and **H**) magnification. The number of inflammatory cells (examples are encircled) were increased in the tumor on Day 31 (**H**) as compared to the pretreatment sample (**E**).

Of the ten tumor biopsies taken before entry into the study, two were diagnosed as grade 1 STS, six as grade 2 STS, and two as grade 3 sarcoma. Additional histology findings are included in Table 3. In the five dogs treated with MYXVΔserp2 intratumorally, biopsies taken on Day 4 were diagnostic for three dogs. All three diagnostic Day 4 biopsy samples contained low numbers of inflammatory cells within the tumor. Biopsies taken on Day 14 also were diagnostics for three of five dogs. Mild mononuclear inflammation was observed within two of the three diagnostic samples. At the end of the study, three of the five dogs treated with intratumoral MYXVΔserp2 had their limb amputated and one had his tail amputated to completely remove the tumor. Histopathology of these lesions indicated that the tumors were completely excised. In contrast, microscopic evaluation of the tumors surgically removed from the five patients treated with MYXVΔserp2 post-operatively confirmed that complete tumor removal was not achieved in any of the dogs. Representative histologic images from Dog 5 are shown in Figure 3.

### 3.5. Peripheral Blood Leukocyte Subsets

**Figure 4:**
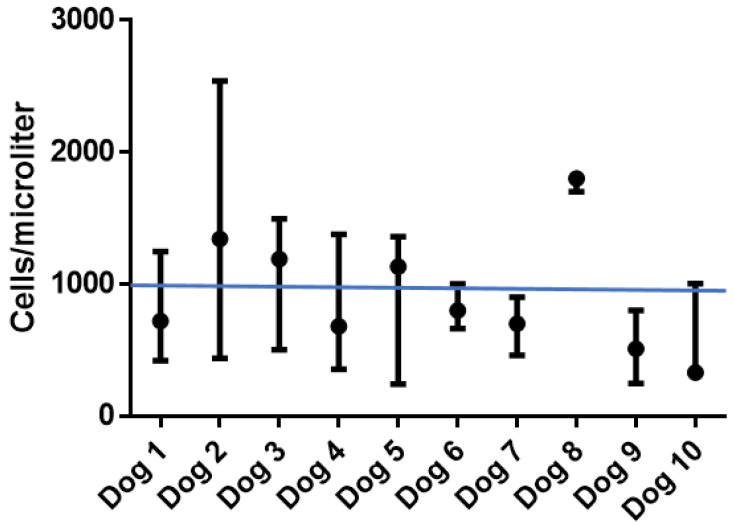
Variation in lymphocytes/microliter of blood. Circles indicate the median and error bars indicate the range of lymphocytes/microliter observed for each dog during the course of the study. The straight line at 1000 cells/microliter represents the low value of the reference interval for lymphocyte concentrations in healthy dogs.

As mentioned previously, 9/10 (90%) cancer patients in this study had decreased numbers of lymphocytes on at least one evaluation of their complete blood counts (Figure 4). Lymphocytes and monocytes were further characterized using flow cytometry. There were no significant changes in the percentages of cell subsets in individual dogs during the course of the study.

### 3.6. Neutralizing Antibody

Anti-MYXV antibodies were not observed in any of the dogs on Day 0. In the five patients given one intratumoral injection of MYXVΔserp2 on Day 0, neutralizing antibodies were detected in 0/5 dogs on Days 14 and 21. In the five patients treated post-operatively on Days 0 and at suture removal, samples were processed for PRNT from Dogs 6 and 7 on Days 0, 14, and 28; Dog 8 on Days 1, 18, and 32; Dog 9 on Days 14 and 28; and Dog 10 on Days 0, 15, and 28. Neutralizing antibodies were noted in 2/5 dogs at Day 28.

## 4. Discussion

This is the first report of the safety of oncolytic poxvirus treatment in dogs with cancer. Close monitoring of patient health using several physical and biological parameters revealed no significant detrimental effects of subcutaneous injection of MYXVΔserp2. Two recent studies using oncolytic viruses in dogs with cancer also have indicated that treatment with an oncolytic virotherapeutic can be safe. An oncolytic reovirus (REOLYSIN^®^) was shown to be safe when administered intratumorally (*n* = 10) or intravenously (*n* = 9) to dogs with various types of cancer [28]. Likewise, minimal AE (transient fever (*n* = 10) and hepatotoxicity (*n* = 1)) were observed following intravenous administration of a recombinant vesicular stomatitis virus to 10 dogs with spontaneous cancers [29]. Data from both studies suggested partial response to treatment in a small subset of patients.

A single intratumoral injection of 10^6^ pfu of the poxvirus MYXVΔserp2 did not alter peripheral blood leukocyte subsets or induce formation of detectable MYXV neutralizing antibodies in the dogs included in this study. Post-operative injection of two doses of 5–10 × 10^6^ pfu MYXVΔserp2 induced anti-MYXV antibodies in 2/5 (40%) dogs. Measurable anti-viral antibody responses have been documented in most people enrolled in clinical trials using oncolytic vaccinia viruses, but antibody response to tumor antigens expressed by oncolytic poxviruses are more variable [72,73,74,75,76,77]. One publication observed a correlation between survival and formation of antiglycan antibodies induced by oncolytic virus treatment [78]. Although pre-existing antibodies against poxviruses contribute to more rapid clearance of virus during an active infection, they have not been shown to negatively affect response to oncolytic therapy [74,79].

For oncolytic virotherapy to be effective, development of a strong cell-mediated immune response is thought to be important due to the ability of CD8^+^ T cells and natural killer cells to directly lyse virus-infected tumor cells [80,81,82,83,84]. Inflammatory infiltrates were minimal in tumor biopsies collected Days 4 and 14 after injection of 10^6^ pfu of MYXVΔserp2. The presence of low numbers of inflammatory leukocytes in soft tissue sarcomas is not uncommon and was unlikely to be a consequence of MYXVΔserp2 treatment. In support of this conclusion, intratumoral viral DNA was not detected in the three patients that an adequate sample was collected from at Day 4. We suspect that a single MYXV∆serp2 injection may be cleared by an innate response before it can elicit a strong cellular or humoral immune response in most dogs. For this reason, further evaluation of intratumoral leukocyte subsets using immunohistochemistry was not pursued.

Slight increases (≤0.6 cm) in tumor diameter were observed in two patients given an intratumoral injection of MYXVΔserp2, while decreases in tumor diameter were measured in three dogs. Clinically, the changes in tumor diameter in four of the dogs were subtle. The VCOG considers changes in tumor diameter <1 cm to be non-measurable by caliper measurement [70]. One patient had a visibly evident 2.7 cm reduction in tumor diameter suggestive of a positive response to MYXVΔserp2 treatment. Variability in patient response to oncolytic virotherapy has been documented in canine and human clinical trials [28,29,85,86,87,88]. An important goal in the field of cancer therapy is to determine which individuals will benefit from specific types of treatment before treatment is initiated. Studies of oncolytic virotherapeutics in mice often do not reveal variable response to treatment. By studying treatment responses of spontaneous tumors in dogs, it is possible that the causes of treatment variability will be elucidated enabling researchers to predict which patients will benefit from oncolytic virotherapy.

Five dogs were recruited into the second arm of this study to determine the safety profile of larger and repeated doses of MYXVΔserp2. We hypothesized that virus injected around a surgical site would infect and eliminate or suppress growth of residual tumor cells in these patients. This hypothesis is supported by a study in cats with vaccine-associated sarcomas which reported decreased tumor recurrence in patients treated post-operatively with recombinant poxvirus expressing interleukin-2 [89]. Additional studies using MYXVΔserp2 post-operatively in dogs with STS are ongoing to determine if tumor regrowth is reduced in dogs that have received this safe anti-cancer viral treatment. We are also exploring the concurrent use of MYXV∆serp2 and an immunotherapeutic to determine if combination therapy will improve treatment efficacy in dogs with STS. 

## 5. Conclusions

The safety of MYXVΔserp2 treatment demonstrated by this study and possible efficacy of treatment warrants recruitment of additional canine cancer patients to determine the true utility of MYXVΔserp2 cancer therapy.

## Figures and Tables

**Table 1:** Primers designed to detect myxoma virus and canine glyceraldehyde 3-phosphate dehydrogenase (cGAPDH) DNA in canine tissues (blood, urine, feces, tumor biopsies) and on swabs (buccal and tumor surface).

Target Gene Sequence	Technique	Forward Primer Sequence	Reverse Primer Sequence	Template Length (Base Pairs)	Annealing Temperature (°C)
Myxoma virus M135R-M136R	Standard PCR	5′-CGA GAA TTC CAC CTG TGT ATG TT-3′	5′-CCA TGT ACA ATA ACA CAC AGT TCG G-3′	1164	52
Myxoma virus M033R-M034L	Standard PCR	5′-CAC CCT CTT TAG TAA AGT ATA CAC C-3′	5′-GAA ATG TTG TCG GAC GGG-3′	818	52
Myxoma virus M033R	Droplet Digital PCR	5′-CGC CAT CCT TTA CCT AAC GA-3′	5′-CGA CAA AAA TAA CAC CGG GT-3′	94	60
Canine GAPDH	Droplet Digital PCR	5′-GCC CTC AAT GAC CAC TTT GT-3′	5′-TCA GCT ACA GCA ACC AGG TG-3′	69	60

**Table 2:** Patient information and history.

Patient	Age (Years)	Breed	Sex	Pertinent Medical History Prior to Study Enrollment	Study Arm	Largest Tumor Diameter on Day 0 (cm)	Largest Tumor Diameter Median (Range) Day 0 to Day 28 (cm)	Tumor Location	Pre-Treatment Biopsy Diagnosis
Dog 1	10	Mixed	Castrated male	Tumor excisions 1 and 2 years prior Regrowth~1 month	Intra-tumoral	2.9	2.9 (2.6–3.5)	Right elbow	Grade 2 STS, PNST
Dog 2	12	Miniature schnauzer	Castrated male	Chronic renal failure Tumor present~3 months	Intra-tumoral	11.0	10.8 (10.2–11.3)	Left inguinal and perianal areas	Grade 1 STS, myxo-sarcoma
Dog 3	8	Mixed	Castrated male	Tumor excision 1 year prior Regrowth~1 month	Intra-tumoral	4.8	4.6 (4.0–5.0)	Left elbow	Grade 3 Undiffer-entiated sarcoma
Dog 4	11	Italian grey-hound	Castrated male	Tumor present~1 month	Intra-tumoral	7.5	7.6 (7.2–7.8)	Tail base	Grade 1 STS, PNST
Dog 5	10	Shetland sheepdog	Castrated male	Tumor present~1 year	Intra-tumoral	14.1	12.1 (10.0–15.3)	Right caudal brachium	Grade 2 STS, PNST
Dog 6	12	Mixed	Spayed female	Tumor excision~4 months prior Regrowth~5 months	Post-operative	5 (per lobule)	TE	Multi-lobulated mass dorsal thorax	Grade 3 Undiffer-entiated sarcoma
Dog 7	11	Greyhound	Castrated male	Tumor excision 2 & 6 years prior Regrowth~1 month	Post-operative	3.5	TE	Left lateral meta-tarsus	Grade 2 STS, PNST
Dog 8	15	Labrador retriever	Castrated male	Tumor present 2–3 years	Post-operative	22	TE	Ventral abdomen	Grade 2 STS, myxoid fibro-sarcoma
Dog 9	12	Labrador retriever	Spayed female	Tumor excision 2 years prior Regrowth~1 month	Post-operative	3.5	TE	Right caudo-lateral thorax	Grade 2 STS, PNST
Dog 10	11	Mixed	Spayed female	Tumor present~1 month	Post-operative	6.5	TE	Left flank	Grade 2 STS, PNST

STS = soft tissue sarcoma. PNST = peripheral nerve sheath tumor. TE = tumor excised on Day 0.

**Table 3:** Histology findings in dogs treated with 10^6^ plaque-forming units of MYXVΔserp2 intratumorally.

Patient	Sample	Diagnosis and Comments	Mitotic Index Per 40× Field	Percent Necrosis	Inflammatory Infiltrate
Dog 1	Pre-treatment biopsy	Grade 2 STS, PNST	1	0	Rare
	Day 4 biopsy	No tumor	N/A	N/A	N/A
	Day 14 biopsy	Grade 2 STS, PNST	1	0	None
	Resected tumor	Grade 2 STS, PNST Slight edema	1	0	Rare
Dog 2	Pre-treatment biopsy	Grade 1 STS, myxosarcoma Edema	1	0	Rare
	Day 4 biopsy	No tumor	N/A	N/A	N/A
	Day 14 biopsy	No tumor Granulation tissue	N/A	N/A	N/A
Dog 3	Pre-treatment biopsy	No tumor	N/A	N/A	N/A
	Day 4 biopsy	Grade 3 undifferentiated sarcoma	6	0	Rare
	Day 14 biopsy	No tumor	N/A	N/A	N/A
	Resected tumor	Grade 3 undifferentiated sarcoma Slight apoptosis	2	10	Rare
Dog 4	Pre-treatment biopsy	Grade 1 STS, PNST	< 1	0	Occasional
	Day 4 biopsy	Grade 1 STS, PNST Fibrosis and hemorrhage	<1	0	Mild
	Day 14 biopsy	Grade 1 STS, PNST	<1	35%	Occasional
	Resected tumor	Grade 1 STS, PNST	<1	10%	Occasional
Dog 5	Pre-treatment biopsy	Grade 2 STS, PNST	1	5%	Mild
	Day 4 biopsy	Grade 2 STS, PNST	1	10%	Occasional
	Day 14 biopsy	Grade 2 STS, PNST	1	5%	Mild
	Resected tumor	Grade 2 STS, PNST	1	50%	Moderate

STS = soft tissue sarcoma. PNST = peripheral nerve sheath tumor.

## Appendix A

Veterinary oncologists or patients’ owners provided information about the health of the patients on a daily basis. Data collected from all patients were assessed using the VCOG-CTCAE grading system. Grade 2 and higher AE are listed in Table A1. Caregivers were asked to indicate the severity of the dogs’ clinical signs (Table A2).

**Table A1:** Metabolic/laboratory adverse events graded ≥ 2 (using Veterinary Comparative Oncology Group criteria) that occurred in dogs treated with MYXVΔserp2. Day 0 data were evaluated prior to MYXVΔserp2 treatment. Patients were monitored for at least 28 days after the first injection of MYXVΔserp2.

Patient	Treatment	Decreased Hematocrit	Decreased Automated Platelet Count	Increased Urea Nitrogen	Increased Creatinine	Increased Phosphorus	Increased Potassium	Increased Calcium	Increased Alkaline Phosphatase	Increased Creatinine Kinase
Dog 1	10^6^ pfu MYXVΔserp2 Day 0									Grade 2 Days 0, 2
Dog 2	10^6^ pfu MYXVΔserp2 Day 0	Grade 2 Days 2, 6, 14, 21, 28		Grade 2 Day 21	Grade 2 Day 21	Grade 2 Days 0–7, 14, 21 Grade 3 Day 28	Grade 2 Days 21, 28			
Dog 3	10^6^ pfu MYXVΔserp2 Day 0		Grade 2 Day 7							
Dog 4	10^6^ pfu MYXVΔserp2 Day 0									Grade 2 Day 1
Dog 5	10^6^ pfu MYXVΔserp2 Day 0									Grade 2 Day 28
Dog 6	10^7^ pfu MYXVΔserp2 Days 0 and 28							Grade 2 Days −2, 14, 28		
Dog 7 *	5 × 10^6^ pfu MYXVΔserp2 Days 0 and 14									
Dog 8 *	10^7^ pfu MYXVΔserp2 Days 0 and 18									
Dog 9	8 × 10^6^ pfu MYXVΔserp2 Days 0 and 14								Grade 3 Days 0, 14, 28	
Dog 10 *	10^7^ pfu MYXVΔserp2 Days 0 and 14									

* No adverse events > Grade 1 were observed.

**Table A2:** Clinical score sheet filled out daily for dogs treated with intratumoral MYXVΔserp2.

Date:	Largest Tumor Diameter (cm):			Weight (pounds):	
Circle a Score for each Clinical Sign	0 = did not occur. 1 = mild, slightly > normal, occurred once, and no treatment needed. 2 = moderate, 2× > normal, occurred more than once, call 970-297-5000 to discuss signs with a veterinarian. 3 = severe, 3× > normal, treatment needed, call 970-297-5000 to make an appointment to see a veterinarian.				
Discharge from tumor site	0	1	2		3
Pain or licking at tumor site *	0	1	2		3
New masses or wounds detected	0	1	2		3
Excessive panting	0	1	2		3
Vomiting	0	1	2		3
Diarrhea	0	1	2		3
Water intake > 200 mL per pound	0	1	2		3
Increased urination	0	1	2		3
Decreased urination	0	1	2		3
Decreased interest in food	0	1	2		3
Lethargy/Malaise	0	1	2		3
Anxiousness/Anxiety	0	1	2		3
Increased vocalization	0	1	2		3
Other observations:					

* Prevent licking at the tumor site by using an E-collar or covering the lesion with a t-shirt or bandage.
